# Antibody-driven capture of synaptic vesicle proteins on the plasma membrane enables the analysis of their interactions with other synaptic proteins

**DOI:** 10.1038/s41598-019-45729-4

**Published:** 2019-06-25

**Authors:** Katharina N. Richter, Christina Patzelt, Nhu T. N. Phan, Silvio O. Rizzoli

**Affiliations:** Institute for Neuro- and Sensory Physiology, Center for Biostructural Imaging of Neurodegeneration, University Medical Center Göttingen, Göttingen, Germany

**Keywords:** Biological techniques, Synaptic vesicle exocytosis, Synaptic vesicle endocytosis

## Abstract

Many organelles from the secretory pathway fuse to the plasma membrane, to exocytose different cargoes. Their proteins are then retrieved from the plasma membrane by endocytosis, and the organelles are re-formed. It is generally unclear whether the organelle proteins colocalize when they are on the plasma membrane, or whether they disperse. To address this, we generated here a new approach, which we tested on synaptic vesicles, organelles that are known to exo- and endocytose frequently. We tagged the synaptotagmin molecules of newly exocytosed vesicles using clusters of primary and secondary antibodies targeted against the luminal domains of these molecules. The antibody clusters are too large for endocytosis, and thus sequestered the synaptotagmin molecules on the plasma membrane. Immunostainings for other synaptic molecules then revealed whether they colocalized with the sequestered synaptotagmin molecules. We suggest that such assays may be in the future extended to other cell types and other organelles.

## Introduction

The question of whether cellular proteins are able to interact or colocalize with each other has been solved by a multitude of biochemical assays, ranging from co-immunoprecipitation, co-fractionation or other gel- and blot-based binding assays^1,2^ to optical methods such as fluorescent resonance energy transfer (FRET^3^) or bimolecular fluorescence complementation^4^. These assays have been employed to determine whether two proteins can interact, either directly or indirectly, at the cell and tissue level. The optical methods also can determine whether the molecules interact in particular cell areas. However, it is difficult to test whether the interaction takes place in a specific cell compartment, especially for molecules that are shared between multiple, densely packed compartments.

This issue is especially important for proteins from organelles of the secretory pathway. Many such organelles, including different types of endosomes, secretory vesicles, or carrier vesicles, fuse frequently to the plasma membrane (exocytosis), to deliver either soluble or membrane-bound cargoes. The organelle molecules are afterwards gathered and are endocytosed, resulting in the reformation of the organelle. How do the molecules colocalize and interact with each other during this entire process? As many of the steps are short-lived, and as the organelles are often found in close vicinity of the plasma membrane, it has been difficult to analyze them. Organelles can be isolated and purified^5,6^, and the interactions between the molecules can then be tested by biochemical tools^7,8^, but this approach does not reveal the potential colocalization of the proteins during the steps between exocytosis and endocytosis, when the organelle molecules are fused to the plasma membrane.

The optical methods mentioned above could in principle be employed, but they generally do not offer the resolution needed to differentiate between molecules on the plasma membrane, or molecules on organelles that are docked to the plasma membrane. Super-resolution imaging could be performed to address this^9^, but one would still have difficulties differentiating between membrane-bound and organelle-bound molecules. To address this issue, we decided to generate an assay in which we immobilize or sequester the proteins of interest on the plasma membrane, and we then verify the colocalization with other proteins by super-resolution imaging. The main advantage of this assay is that there is no uncertainty about the molecule positions – they need to be on the plasma membrane, where the sequestration system maintains them.

To perform this, we identified recently exocytosed molecules using mouse monoclonal antibodies. We then immobilized them on the plasma membrane by binding aggregates of secondary antibodies to the monoclonal antibodies. The aggregates were too large to be endocytosed, and therefore forced the recently exocytosed molecules to remain on the plasma membrane. To verify whether other proteins colocalized with the immobilized ones, we resorted to immunostainings and stimulated emission depletion (STED) microscopy^10^. The preferred biological system was the synaptic bouton, where synaptic vesicles regularly fuse to the plasma membrane to release their neurotransmitters, and are then endocytosed and refilled with neurotransmitter^11,12^. This system relies on accurate exo- and endocytosis, and was especially convenient for the present investigation, since numerous imaging tools are available for the synapse, including a variety of antibodies for synaptic proteins (e.g.^13,14^). Importantly, monoclonal antibodies against the luminal (intravesicular) domain of a well-studied transmembrane protein, the calcium sensor synaptotagmin 1, have been characterized for more than two decades^15,16^. These antibodies are highly specific, and have already been used in a large number of studies of neuronal physiology. For example, they have been shown to reveal the surface population of this molecule with high precision^17,18^, both in conventional microscopy studies and in super-resolution investigations^10,19^. Linking the antibodies to pH-sensitive fluorophores enabled accurate readings of both exo- and endocytosis^20^. Using quantum dots linked to the antibodies allowed endocytosis investigations^21^ and even enabled the analysis of single exocytotic events^22,23^. The antibodies can be taken up by vesicles recycling both during stimulated activity and spontaneously^24^, and do not seem to affect the vesicles in the short- or medium-term (a few days^25^), although a rabbit polyclonal antibody against the same target has been shown to perturb synaptic activity^26^.

We immobilized synaptotagmin 1 molecules on the plasma membrane, using the monoclonal antibodies mentioned above, and we investigated their colocalization with several other synaptic proteins. We targeted specifically newly exocytosed molecules, rather than all synaptotagmin 1 molecules from the plasma membrane. We found that plasma membrane-immobilized synaptotagmins colocalized with many other proteins of the exo- and endocytosis pathway, but not with a classical marker of sorting endosomes, EEA1. We suggest that similar approaches could be used in the future to investigate the proteome of fused organelles of the secretory pathways.

## Results

### Surface epitope blocking enables the investigation of recently-exocytosed molecules

**Figure 1:**
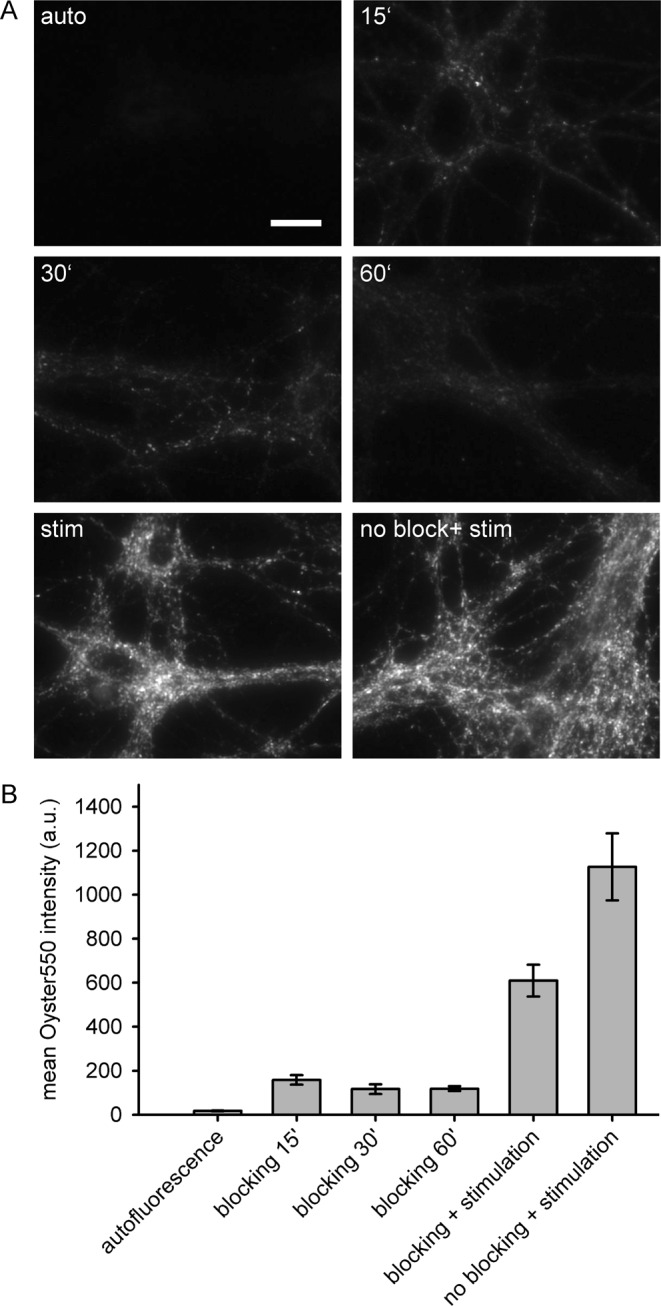
The application of synaptotagmin antibodies onto neuronal cultures blocks the surface-resident synaptotagmin epitopes. (**A**) The surface synaptotagmin epitopes of live neurons were blocked with a 1:20 dilution of anti-synaptotagmin antibody (from a starting concentration of 1 mg/ml) for 15, 30 and 60 minutes. The remaining non-blocked synaptotagmin epitopes were visualized by a subsequent incubation with an Oyster550-conjugated anti-synaptotagmin antibody. The Oyster550 signal intensity was compared to signal intensities from an autofluorescence control (consisting of cultures not exposed to any antibody) and to cultures that were either blocked for 30 minutes and then stimulated in presence of Oyster550-conjugated antibodies, or stimulated without any prior blocking procedure. Scale bar = 20 μm. (**B**) We analyzed the Oyster550 intensities of the different experiments shown in panel A. The best blocking efficiency is achieved with an incubation time of at least 30 minutes. The bars represent means ± SEM (N = 10 frames for each condition).

We used as our cellular model primary cultured hippocampal neurons, allowed to mature for about 14 days *in vitro*^13,21,25^. To specifically target synaptotagmin molecules from recently-exocytosed molecules, we aimed to first eliminate the antigenicity of the synaptotagmin molecules resident on the plasma membrane. This can be performed by incubating the neurons with synaptotagmin antibodies for a few minutes, which block the synaptotagmin epitopes (Fig. 1). Subsequent stimulation results in the exocytosis of new synaptic vesicles, thereby exposing on the plasma membrane new synaptotagmin molecules, which can be revealed by incubation with different antibodies.

We assessed the efficiency of the blocking procedure by visualizing the non-blocked synaptotagmin via labeling with Oyster550-conjugated anti-synaptotagmin antibodies (Fig. 1A). Different blocking durations were tested (with the neurons incubated with the blocking antibodies in presence of tetrodotoxin, TTX, to inhibit neuronal signaling and synaptic vesicle recycling) and the fluorescence intensity of the stainings was compared to a non-blocked and stimulated control. A quantification of the blocking efficiencies (Fig. 1B) demonstrated that the wide majority of the membrane-resident epitopes could be blocked by incubations of ~30 minutes with synaptotagmin antibodies.

### The general organization of the assay

**Figure 2:**
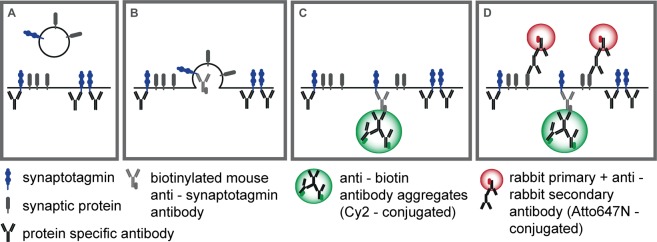
A general overview of the assay designed to retain synaptotagmin molecules on the plasma membrane. Surface synaptotagmin epitopes are blocked with anti-synaptotagmin antibodies against the luminal domain (**A**). After washing off unbound antibodies, newly exocytosed synaptotagmin is bound by biotinylated anti-synaptotagmin antibodies (**B**). After an additional washing step, antibody aggregates directed against biotin (green) are applied (**C**). Neurons are washed, fixed and permeabilized, prior to the immunostaining of additional proteins (grey) by respective antibodies and Atto647N-conjugated secondary antibodies (red; **D**).

The procedure followed here is summarized in Fig. 2, and consists of the following. First, surface synaptotagmin 1 epitopes resident on the plasma membrane were blocked with anti-synaptotagmin 1 antibodies in the presence of TTX (Fig. 2A). After washing off the blocking antibodies, neurons were incubated with biotinylated anti-synaptotagmin 1 antibodies, which bind to the newly exocytosed synaptotagmin (Fig. 2B). For exocytosis we relied on the spontaneous activity of the cultures, which takes place in bursts releasing ~3–6 vesicles per synaptic bouton, at ~0.1 Hz^25^. After another washing step, we applied aggregates of goat anti-biotin antibodies and secondary antibodies against goat antibodies (Fig. 2C). These should bind to the biotinylated synaptotagmin antibodies, and should sequester the molecules on the plasma membrane, by inhibiting compensatory endocytosis (Fig. 2C).

The samples were then fixed, and various synaptic proteins were immunostained, using the respective antibodies and secondary antibodies conjugated to Atto647N (Fig. 2D). As there is no blocking procedure for the investigated synaptic proteins, immunostaining labeled all available epitopes in the synapse, both on the membrane and in the synaptic cytosol. The samples were finally imaged using confocal and STED microscopy.

### Biotinylated synaptotagmin antibodies detect the recently exocytosed molecules, and are in turn revealed by secondary antibody aggregates

**Figure 3:**
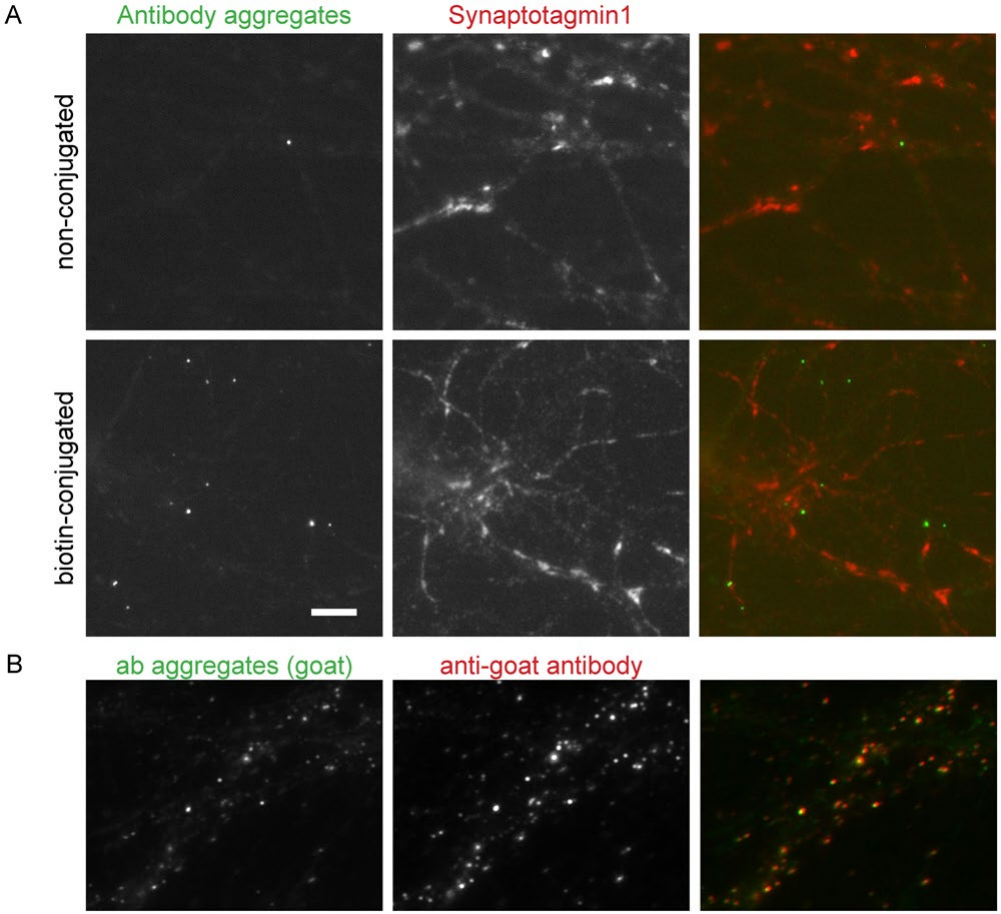
Antibody aggregates detect biotinylated synaptotagmin antibodies in a specific fashion. (**A**) Live neurons were incubated with non-conjugated anti-synaptotagmin antibodies (upper panels) or biotin-conjugated anti-synaptotagmin antibodies (lower panels). Subsequent incubation with freshly prepared antibody aggregates shows specific binding to biotin-labeled cultures, and very little non-specific binding. For better orientation, neurons were additionally labeled for synaptotagmin using an Atto647N-conjugated anti-synaptotagmin antibody restricted to its cytoplasmic domain. Scale bar = 5 μm. (**B)** To determine whether the antibody aggregates were endocytosed, or were still exposed on the surface, we incubated the cultures, after fixation, with mouse anti-goat antibodies conjugated to a different fluorophore. Virtually all antibody aggregates were detected (98% average colocalization, as observed from 3 independent experiments). Scale bar = 5 µm.

To test whether antibody aggregates can indeed sequester synaptotagmin molecules on the plasma membrane, we first constructed them *in vitro* by incubating polyclonal secondary antibodies with goat anti-biotin antibodies in PBS, at 37 °C, overnight. The secondary antibodies were labeled fluorescently, to ensure that the aggregates can be detected in optical microscopy. The aggregates were centrifuged for 30 minutes at maximum speed in a tabletop centrifuge, to remove single antibodies. The resuspended pellet was sonicated (in a sonication water bath) and was centrifuged again for 1–2 min in order to remove extremely large aggregates. The remaining solution was then applied on neurons whose synaptotagmin epitopes had been blocked, and whose newly exocytosed vesicles had been already labeled using biotinylated synaptotagmin antibodies (Fig. 3A). Optimal results were obtained using a donkey anti-goat antibody: the aggregates bound neurites specifically, and were only rarely found on the coverslips when the biotinylated antibodies were omitted (Fig. 3A). Interestingly, chicken anti-goat antibodies could not be used in this assay, since they did not form antibody aggregates, possibly due to a limited polyclonality.

We also verified whether the antibody aggregates indeed remained on the surface of the plasma membrane, or whether they were endocytosed in these cultures. We revealed the antibodies using mouse anti-goat antibodies (with a different fluorescent label than the antibody aggreagates), in the absence of permeabilization, to prevent the detection of already internalized antibodies. We found that virtually all antibody aggregates were readily detected (98% colocalization, Fig. 3B).

### A colocalization analysis for several synaptic proteins

The localization of several proteins was then investigated in relation to the recently-exocytosed synaptotagmin, by immunostaining them on the cultures decorated with antibody aggregates (see Supplementary Table S1 for further information on the antibodies used for protein labeling). All of the proteins have been immunolabeled with rabbit polyclonal antibodies (with the exception of AP2, labeled using a rabbit monoclonal antibody), and Atto647N-conjugated anti-rabbit antibodies. We also determined whether the anti-rabbit antibodies produced any cross-reactivity. No measurable cross-reactivity was observed, other than the expected autofluorescence of the cell cultures (Supplementary Fig. S1). The antibody aggregates did not change in size during the incubation time (Supplementary Fig. S2).

**Figure 4:**
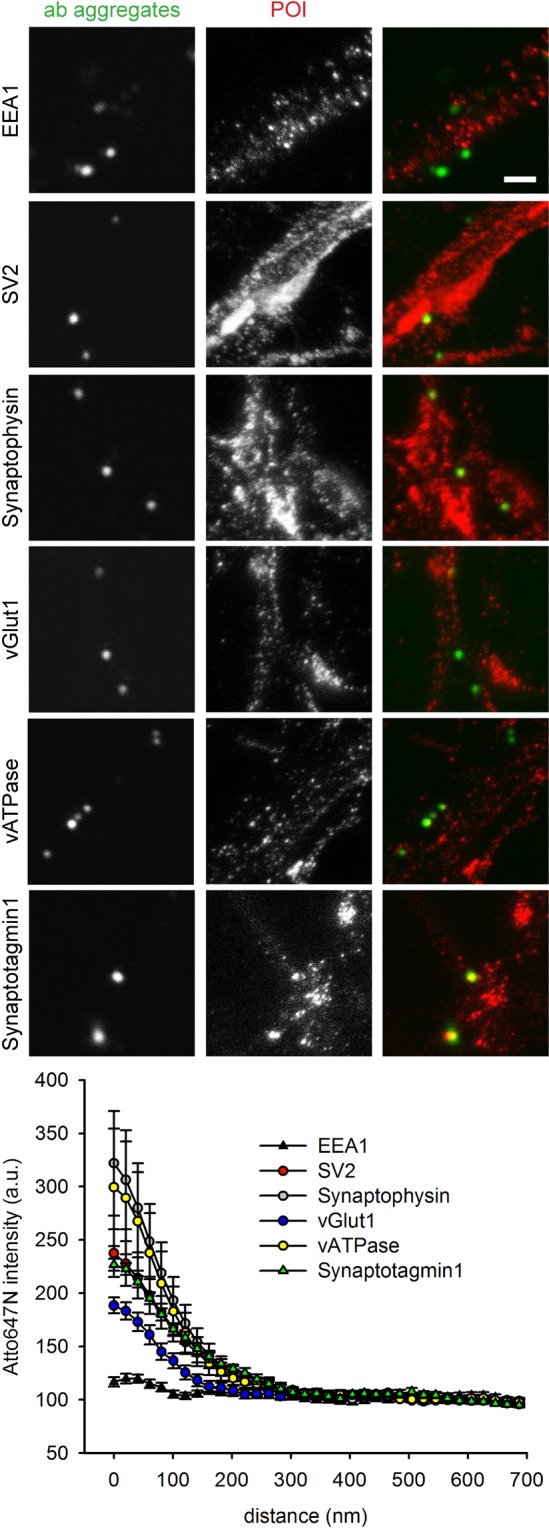
STED and confocal images of the co-localization studies for synaptic vesicle proteins. The neurons were immunostained following the protocol indicated in Fig. 2. Cy2-conjugateded antibody aggregates (green spots) were imaged in confocal mode, while Atto647N-labeled proteins (red spots) were imaged in STED mode. Scale bar = 1 μm. The graphs show an analysis of the colocalization of the proteins, in the form of the Atto647N intensity as a function of distance from the center of the antibody aggregate spots. N = 5 (vGlut1/2) and 4 (all other conditions) independent experiments. The error bars show the SEM from 4–5 independent experiments.

**Figure 5:**
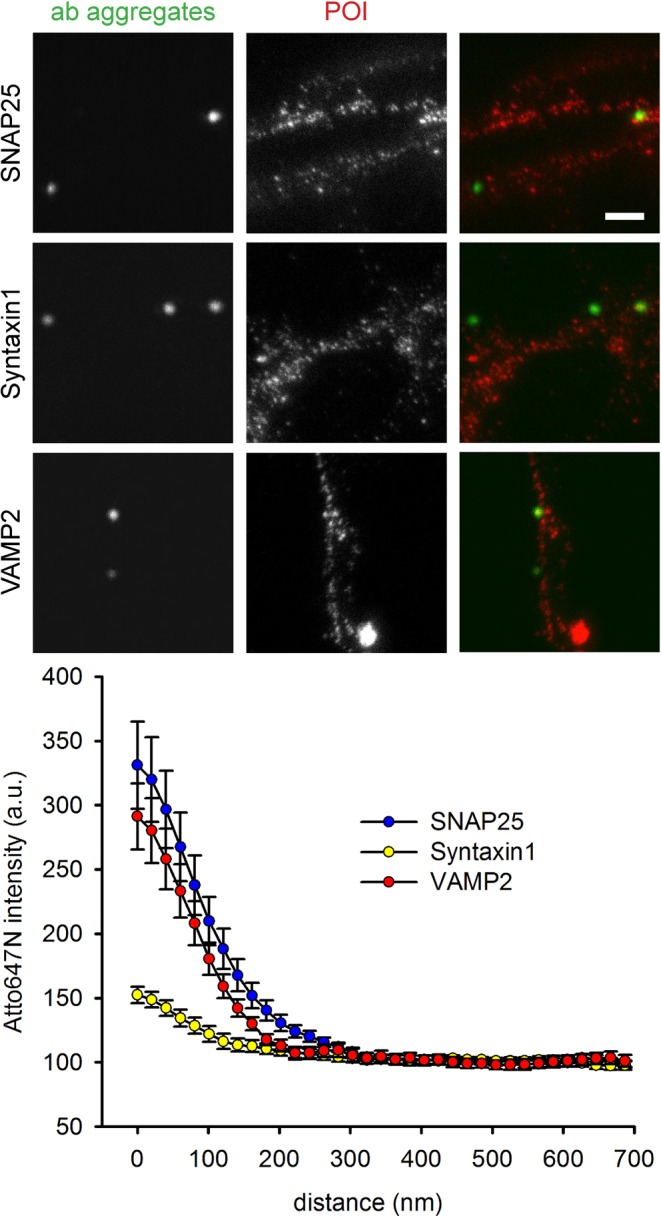
STED and confocal images of the co-localization studies for SNARE proteins. The neurons were immunostained following the protocol indicated in Fig. 2. Cy2-conjugateded antibody aggregates (green spots) were imaged in confocal mode, while Atto647N-labeled proteins (red spots) were imaged in STED mode. Scale bar = 1 μm. The graphs show an analysis of the colocalization of the proteins, in the form of the Atto647N intensity as a function of distance from the center of the antibody aggregate spots. N = 3 (VAMP2) and 4 (all other conditions) independent experiments. The error bars show the SEM from 3–4 independent experiments.

**Figure 6:**
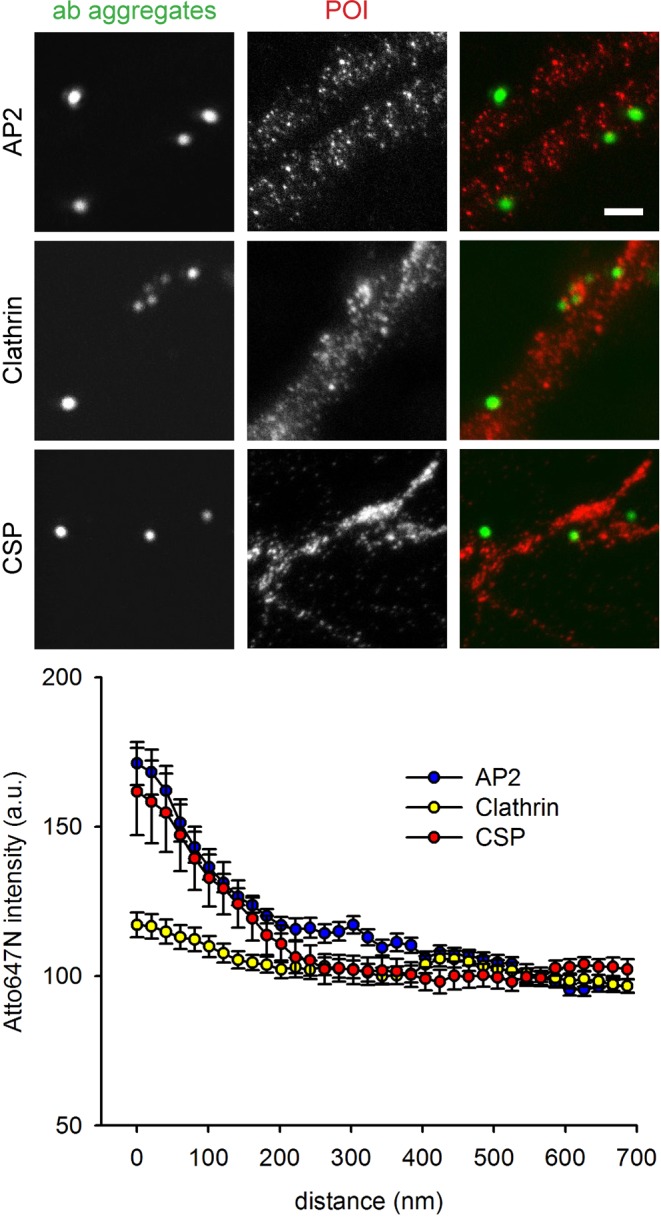
STED and confocal images of the co-localization studies for endocytosis co-factors. The neurons were immunostained following the protocol indicated in Fig. 2. Cy2-conjugateded antibody aggregates (green spots) were imaged in confocal mode, while Atto647N-labeled proteins (red spots) were imaged in STED mode. Scale bar = 1 μm. The graphs show an analysis of the colocalization of the proteins, in the form of the Atto647N intensity as a function of distance from the center of the antibody aggregate spots. N = 3 (Clathrin), 5 and 4 (all other conditions) independent experiments. The error bars show the SEM from 3–5 independent experiments.

We followed this up with the analysis of 12 proteins, representing different phases of synaptic vesicle recycling. First, we targeted a number of synaptic vesicle proteins, including synaptotagmin itself (which also serves as a positive control), the synaptic vesicle markers synaptophysin and SV2, the neurotransmitter transporter (vGlut), the proton pump that is involved in refilling the synaptic vesicle with neurotransmitter (vATPase), and EEA1 (early endosomal autoantigen 1), which is a well-known marker of early endosomes, where it mediates vesicular transport^27^, but is not known to localize in the neuronal plasma membrane, and therefore served as a negative control (Fig. 4). Second, we immunostained the plasma membrane exocytosis SNARE proteins, syntaxin 1 and SNAP25^28^ and the vesicular exocytosis SNARE molecule synaptobrevin2/VAMP2^12,29^ (Fig. 5). Third, we analyzed an exocytosis cofactor protein that is strongly associated to synaptic vesicles, but lacks transmembrane domains, cysteine-string-protein-alpha (CSPalpha^25^). Fourth, we immunostained two endocytosis cofactors, clathrin and AP2^12,29^ (Fig. 6).

**Figure 7:**
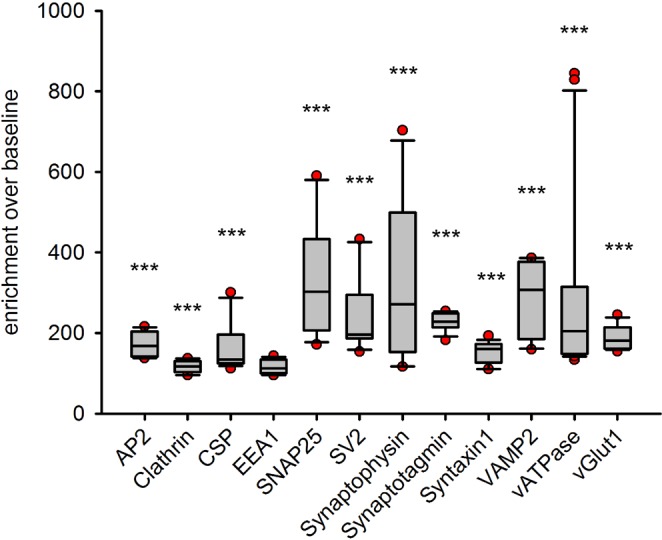
An overall analysis of protein enrichment in the vicinity of the antibody aggregates. We analyzed the enrichment, over baseline, of each protein, and compared them to their baselines, relying on a Kruskal-Wallis test to determine significant differences at the level of the entire dataset, followed by Mann-Whitney tests, and a Bonferroni correction for multiple testing. These demonstrated that the EEA1 enrichment was not statistically significant, but all others were (all Bonferroni-corrected P values < 0.0001). Box plots show the medians with 25^th^ and 75^th^ percentiles of the data. The bars represent the 10^th^ and 90^th^ percentiles and the dots show outliers. The values are taken from Figs 4–6, and represent the first (leftmost) data points in the intensity distributions, normalized to the respective baselines (all data points, from all experiments, are shown here, while only averages are shown in Figs 4–6).

We analyzed the colocalization of the different proteins to synaptotagmin 1 by measuring the fluorescence intensity of the different immunostainings in relation to distance from the center of the antibody aggregates. As the aggregates were found at large distances to each other, we analyzed them by confocal imaging. This was preferable to super-resolution imaging, since the high signal-to-noise ratio of the confocal images enabled us to determine the position of the center of mass of the aggregate signals with high precision; a STED image of the same spots would not provide any further information on the center of mass^5^. This type of analysis has been used in the past for such datasets (for example^30,31^), and is similar to the analysis of Ripley’s K function^32^ (see Supplementary Fig. S3 for more details). Overall, this analysis demonstrated that all proteins colocalized with synaptotagmin to different extents, with the exception of EEA1 (Fig. 7).

To obtain further insight into the different behaviors of the proteins, we tested the relation of the protein enrichment to several biological parameters. First, we observed that the recruitment of soluble proteins was substantially below that of membrane proteins (p = 0.016; Supplementary Fig. S4,A). The number of transmembrane domains did not seem to cause a significant difference (Supplementary Fig. S4B). Interestingly, we found a significant correlation between the levels of recruitment of the proteins and the respective mRNA amounts (as measured in the rodent brain in the past^33^), although no correlation could be found with protein abundances (again measured in the rodent brain in the past^34^, in purified synapses^13^, or in hippocampal cultures^35^), as shown in Supplementary Fig. S4C,D. Finally, the recruitment of proteins to synaptotagmin molecules seemed to be more difficult for larger proteins, but the trends observed were not significant (p = 0.07–0.96; Supplementary Fig. S4E,F).

## Discussion

We generated here a tool for the analysis of the colocalization of different synaptic proteins with plasma membrane-sequestered synaptotagmin molecules. The latter are bound by biotinylated synaptotagmin 1 antibodies, which are prevented from endocytosing by binding to aggregates containing goat anti-biotin antibodies. The samples can then be fixed and immunostained, and the colocalization of various proteins with the antibody aggregates can be analyzed.

As mentioned in the Introduction, the synaptotagmin antibodies have been thoroughly characterized in past works^10,15–25,36–38^. Synaptotagmin is one of the few vesicle molecules that could be used for this assay, since only few antibodies for the lumenal domains of major synaptic vesicle proteins exist, as for the GABA transporter^39^. Synaptophysin may eventually make a good target, since one polyclonal antibody^40^ could be used in such experiments in fixed cells^21^, albeit not in live cells, implying that one may be able to generate synaptophysin antibodies for live-cell work in the future.

An alternative would be the use of GFP-tagged vesicle proteins, in which the GFP moiety (or a pH-sensitive variant, pHluorin) is present in the lumen of the vesicle^41^. Many studies have employed this tagging opportunity, for multiple vesicle proteins^21,42–45^. Not only antibodies, but also smaller probes are available for binding to GFP, such as nanobodies^46^, which enable high-precision imaging investigations by live super-resolution imaging, such as subdiffractional tracking of internalized molecules (sdTIM^47^). While GFP-tagged vesicle proteins seem to function well, some concerns about their proper targeting have been raised^48,49^, albeit the problems may be connected more to the levels of overexpression than to the tag.

We tested here the colocalization of membrane-bound synaptotagmin with several proteins, and we found that in most cases a strong colocalization could be measured. This observation is, of course, expected for synaptotagmin itself, but is an open question for the other proteins. The organization of fused synaptic vesicles is still under discussion, with two models being repeatedly discussed in the literature^12,50,51^. First, the vesicle proteins may maintain a vesicle-like arrangement, by binding to each other, and forming a patch of molecules on the plasma membrane^10^. Second, the vesicles do not bind each other in the plasma membrane, and diffuse away from each other. The endocytosis machinery then retrieves them separately, and may need to re-assemble synaptic vesicles in a compartment similar to sorting endosomes.

The observations presented here are in better agreement with the first hypothesis, and are also in agreement with observations from previous super-resolution investigations of fused synaptic vesicles, which suggest that patches of multiple molecules are present on the plasma membrane (see for example^19,21,25,52^), or with biochemical and cell biology investigations in which strong interactions between synaptic vesicle proteins have been described^7,48,53–55^. These observations cannot discount the possibility that a limited, but not negligible, loss of molecules from the fused vesicle patches does take place. The binding of the endocytosis machinery components to the recently exocytosed components (Fig. 6) may limit this diffusion^56^, and may keep it to levels that would enable vesicles to maintain their composition and their protein-protein interactions over time^25^.

At the same time, these observations also raise an interesting additional point. It is not necessarily expected that the plasma membrane SNAREs (syntaxin 1, SNAP25) enrich at the sites of fused vesicles. SNAREs need to be disentangled from each other before budding of vesicles from larger membranes^57^, and therefore one would expect that these molecules would leave the sites of the fused vesicles rapidly (as suggested from recent live single-molecule investigations^58^). At the same time, vesicles preferentially dock and fuse next to regions of high SNARE density^59^, which implies that some association with these molecules is expected. Nevertheless, the strong colocalization observed here (Fig. 5) is unlikely to be a simple association to SNARE protein clusters, since the plasma membrane clusters of syntaxin 1 and SNAP25 colocalize only to a limited extent^60,61^. Synaptotagmin could be expected to colocalize randomly with one of the proteins, but not with both, which therefore implies that more specific interactions of syntaxin 1 and SNAP25 to the fused vesicle proteins may take place.

However, some caveats of this technique are also apparent. First, synaptic vesicles are small organelles, and therefore they are expected to have problems in retrieving antibody aggregates. This may not be the case when larger organelles are investigated. These may be able to endocytose the aggregates, thereby nullifying the entire assay. This could be circumvented by using beads of defined size, which would target the molecules of interest. We attempted to perform this here, using streptavidin-coated beads as a detection system for the biotinylated synaptotagmin antibodies (Supplementary Fig. S5), but we observed extensive non-specific binding. Such experiments will therefore probably require substantial optimization.

Second, the use of antibodies as protein-detection tools has its own limitations. Antibodies only recognize a proportion of the targets that can be detected in a cell by smaller affinity tools, such as aptamers or nanobodies^62,63^. They are also bivalent, and therefore may induce a certain level of clustering of protein targets^64^. Assays based on smaller affinity tools^65^ would be preferable, for the initial detection of the proteins. Nevertheless, it is the unwanted characteristics of antibodies that are exploited in the rest of the present assay: their ability to bind two targets simultaneously enables them to form the aggregates, and their large size renders the aggregates sufficiently large for interfering with endocytosis. An assay in which antibody aggregates would be applied onto proteins detected by biotinylated nanobodies or aptamers would be therefore ideal as a future tool for this direction of research.

## Methods

### Primary hippocampal cultures

Rat primary hippocampal cultures were prepared as described in^52^. In summary, hippocampi from newborn rats were dissected in HBSS (Life technologies, Gibco cat. #24020-091) and incubated for one hour at 37 °C in DMEM supplemented with 2 mg cystein, 100 mM CaCl_2_, 50 mM EDTA and 25 U papain/mL, under CO_2_ bubbling. Afterwards hippocampi were incubated for 15 min in DMEM supplemented with 5% fetal calf serum, 25 mg BSA and 25 mg trypsin inhibitor. Subsequently trituration in Neurobasal medium was performed (Neurobasal A, with 20% B27 and 10% Glutamax-I, all from Life technologies/Gibco, and with 20 units/mL penicillin and 20 μg/mL streptomycin). Finally neurons were plated in MEM (Life technologies/Gibco) supplemented with 3,3 mM glucose, 10% horse serum and 2 mM glutamine, on Poly-L-Lysin coated coverslips. The medium was exchanged for Neurobasal after one to two hours of plating.

### Blocking surface synaptotagmin epitopes

Surface synaptotagmin was blocked by incubation of living neurons with non-labeled anti-synaptotagmin antibodies (Synaptic Systems, cat. #105 311) that are directed to the lumenal domain of the molecule. Therefore, living neurons were first incubated in 0.5 μM Tetrodotoxin (TTX), for a few minutes at 37 °C, in order to inhibit neuronal activity. Neurons were then incubated with the mouse anti-synaptotagmin antibody (diluted 1:20 in the culturing medium of the respective coverslips and 0.5 μM TTX) for 30 min in a 37 °C incubator. In order to test the efficiency of this surface synaptotagmin blocking, non-blocked synaptotagmin was visualized by incubation with Oyster550-conjugated anti-synaptotagmin antibodies. The fluorescence intensity was compared to the intensity from neurons that were only incubated with Oyster550-conjugated anti-synaptotagmin antibodies while stimulated with a 60 mM K^+^ solution (non-blocked control) and neurons that were surface-blocked, but were also afterwards stimulated in the presence of the Oyster550-conjugated antibody (newly exocytosed synaptotagmin control). The fluorescence intensities were therefore imaged with an Olympus fluorescence microscope and compared with Matlab (The Mathworks Inc., Natick, MA, USA).

### Antibody aggregates

Antibody aggregates consisting of goat anti-biotin (Thermo Scientific, Pierce antibodies cat. #31852) and Cy2-conjugated donkey anti-goat (Dianova cat. #705-225-147) antibodies were prepared by incubating an equal amount of both antibodies in PBS over night at 37 °C in a water bath. Afterwards non-aggregated antibodies were washed off by 30 min centrifugation at 13.2 rpm (in an Eppendorf 5415R centrifuge). The resuspended pellet was sonicated for five minutes (sonication water bath) in order to break large aggregates. The first centrifugation of the antibodies induced very large aggregates that were eliminated from use by centrifuging the resuspended and sonicated pellet, again for 1–2 minutes at 13.2 rpm, and using only the supernatant. Neurons were incubated for 30 min at 37 °C with biotinylated anti-synaptotagmin antibodies, diluted in the neuronal culture medium, followed by 2–3 times washing in Tyrode. The prepared antibody aggregates from the supernatant were diluted 1:10 in the neuronal culture medium, and the neurons were incubated for 10 min at 37 °C. Afterwards neurons were washed 3 times in Tyrode and were used for further immunocytochemistry experiments.

### Immunocytochemistry

Neurons for immunocytochemistry experiments were all fixed with 4% Paraformaldehyde, first 10 min on ice and afterwards another 30 min at room temperature, followed by 2 times 5 min washing in PBS. Quenching was performed by incubating the neurons in 100 mM NH_4_Cl for 20 min. Afterwards the neurons were permeabilized with 0.1% Triton X-100 and were blocked with 2.5% BSA in PBS, 3 times for 5 min. Primary antibodies were applied for 1 h in the dark in a humidified chamber (dilutions in PBS containing 0.1% Triton X-100 and 2.5% BSA according to Supplementary Table S1). Prior to the application of secondary antibodies (also 1 h in the dark), neurons were washed again in PBS containing 0.1% Triton X-100 and 2.5% BSA. After incubation with secondary antibodies (dilutions according to Supplementary Table S1), the neurons were washed in PBS containing 2.5% BSA and in high-salt PBS as well as in PBS. Finally, neurons were embedded in Mowiol, and the samples were kept at 4 °C until imaging.

### Imaging

Epifluorescence imaging of immunolabeled samples was performed using an inverted flucorescence microscope (Olympus IX 71). A 40x (Olympus UPlan FLN, NA 0.75) and a 100x oil objective (Olympus PlanApo, NA 1.45) were used, and signals from different fluorophores were recorded in separate channels using integrated filters of the Olympus microscope. For confocal and STED imaging, a TCS SP5 STED fluorescence microscope from Leica Microsystems GmbH (Mannheim) was used. The samples were imaged with a 100x oil objective (Leica, NA 1.4). For STED imaging the samples were labeled with Atto647N, which was excited by a 635 nm pulsed diode laser and depleted with a Spectra-Physics MaiTai tunable laser at 750 nm. Fluorescence was detected in STED mode using an avalanche photodiode. Atto647N and Cy2 were imaged in confocal mode using photomultipliers and a helium-neon and argon laser. All settings (gain, laser intensity, pinhole size) were kept the same for all images of one sample, in order to be able to compare fluorescence levels in different neurons and areas.

### Data analysis

All analyses were done in Matlab, using self-written routines (macros). For assessing the surface blocking efficiency a macro was used to calculate the mean fluorescence intensity for every image.

In order to correlate the fluorescence of the Atto647N-labeled proteins with that of the Cy2-conjugated antibody aggregates, STED and confocal images were first aligned in Matlab, and all Cy2 spots were automatically selected, using an empirically-determined intensity threshold. Square regions of interest centered on the centers of mass of the Cy2 spots were then selected, with a square breadth and height of ~1400 nm (71 pixels). We generated regions of interest separately for the Cy2 and the Atto647N channels, on the exact same locations indicated by the centers of mass in the Cy2 signal.

The regions of interest were then summed, thereby obtaining summed matrices of 71 × 71 pixels. We summed the regions of interest in the Cy2 channel and in the Atto647N channel separately. The summed matrix in the Cy2 channel always showed a bright image of the conjugated antibody aggregates. The summed matrix in the Atto647N channel presented the signal that colocalized with the conjugated antibody aggregates. If the protein analyzed in this channel colocalized well with the antibody aggregates, then a bright spot was observed in the center of the summed Atto647N matrix. If the colocalization was low, the signal in the center of this matrix was not clearly distinguishable from background. To then analyze this quantitatively, we drew line scans across the summed matrices, starting in the center, and measured the intensity in the Atto647N channel, in relation to distance from the center. The higher this intensity was, in comparison with the baseline away from the center of the matrix, the more the protein correlated with the antibody aggregates. For more details see Supplementary Fig. S3.

### Coupling streptavidin-labeled beads to biotinylated anti-synaptotagmin antibodies

Fluorescent streptavidin-labeled beads were purchased from Life technologies (cat. #F 8780, ex 505/em 515) and had to be coupled to biotinylated anti-synaptotagmin antibodies (Synaptic Systems, cat. #105 311BT) in order to bind synaptotagmin in the plasma membrane. Therefore, the beads were sonicated for five minutes in a sonication water bath and mixed with the anti-synaptotagmin antibodies in Tyrode buffer (beads in a dilution of 1:100 and 1:50; different dilutions of antibodies were tested, and the best dilution for saturating the beads was found to be 1:20). The solution was incubated for 2 h rotating at 4 °C. Afterwards unbound antibodies were washed away by 10 min centrifugation in an Eppendorf 5415R centrifuge (13.2 rpm). After resuspension of the pellet in Tyrode, the solution was sonicated again for a few minutes, frozen in liquid nitrogen and stored at −80 °C until further use.

### Animals

Wild type Wistar rats (*Rattus norvegicus*) for the preparation of primary hippocampal neuron cultures were obtained from the University Medical Center Göttingen. All animals were handled according to the specifications of the University of Göttingen and of the local authority, the State of Lower Saxony (Landesamt für Verbraucherschutz, LAVES, Braunschweig, Germany). All animal experiments were approved by the local authority, the Lower Saxony State Office for Consumer Protection and Food Safety (Niedersächsisches Landesamt für Verbraucherschutz und Lebensmittelsicherheit).

## Supplementary information

Supplementary Information - changes not highlighted

## Data Availability

The datasets generated during and/or analyzed during the current study are available from the corresponding author on reasonable request.
