# Supplementary material for: Antibody-driven capture of synaptic vesicle proteins on the plasma membrane enables the analysis of their interactions with other synaptic proteins
Source: Sci Rep. 2019 Jun 25;9:9231. doi: 10.1038/s41598-019-45729-4 (PMC6592915; doi:10.1038/s41598-019-45729-4)
Supplement: Supplementary file 1 — Supplementary Information - changes not highlighted [file 41598_2019_45729_MOESM1_ESM.docx]

Supplementary Information for

**Antibody-driven capture of synaptic vesicle proteins on the plasma membrane enables the analysis of their interactions with other synaptic proteins**

Katharina N. Richter^1,*^, Christina Patzelt^1^, Nhu T.N. Phan^1^, Silvio O. Rizzoli^1,*^

^1^Institute for Neuro- and Sensory Physiology, Center for Biostructural Imaging of Neurodegeneration, University Medical Center Göttingen, Göttingen, Germany.

*correspondence to: Katharina N. Richter (k.richter1@stud.uni-goettingen.de), Silvio O. Rizzoli (srizzol@gwdg.de)


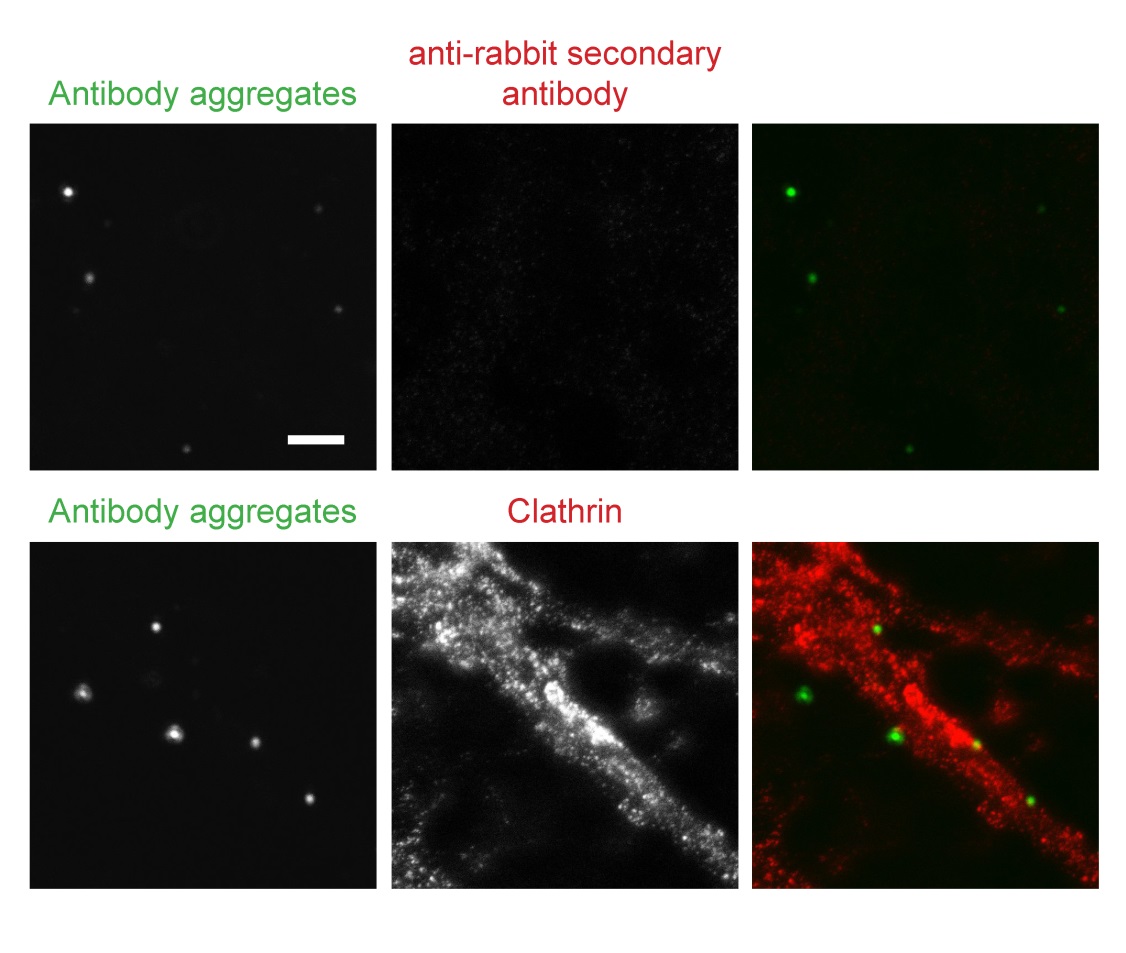


**Supplementary Figure S1. A crossreactivity test for the antibody aggregates and the Atto647-conjugated anti-rabbit antibody in the immunostainings of various synaptic proteins.** Neurons were treated as for immunostaining a protein for co-localization studies, but without incubation with rabbit primary antibodies (upper panel). A comparison of Cy2 and Atto647N signals to the signals from an actual anti-clathrin staining (lower panel) shows no crossreactivity of the antibody aggregates and the anti-rabbit antibody. Scale bar = 2 μm.

**
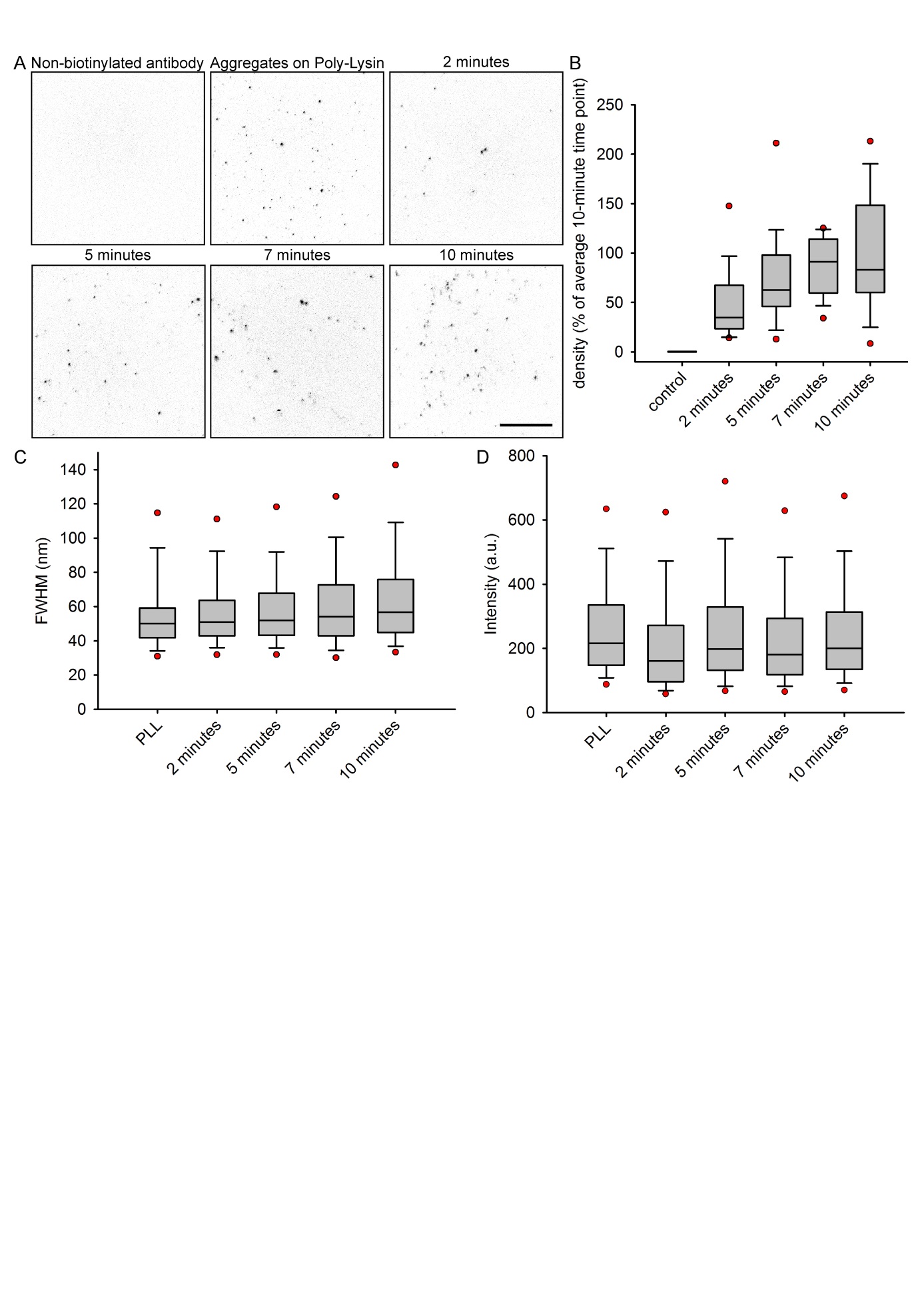
**

**Supplementary Figure S2. An analysis of the antibody aggregates over time. A**, Live neurons were incubated with anti-synaptotagmin antibodies not conjugated to biotin (upper left panel) or with biotin-conjugated antibodies (upper right panel and lower panels). The cultures were then incubated for different amounts of time, as indicated on the panels, with antibody aggregates directed against biotin, prepared as described in Methods, but with Star635P-conjugated donkey anti-goat antibodies (Abberior, cat. # ST635P-1055-500UG), which enables their analysis by STED microscopy. For control purposes, we also incubated coverslips coated with poly-L-Lysine (PLL), without neurons, with the aggregates (upper middle panel). The control cultures lacking biotin-conjugated antibodies were incubated with the aggregates for 10 minutes. Scale bar = 5 µm. **B-C**, We analyzed the images as follows. An automatic, empirically-determined threshold was applied to identify all spots above background (corresponding to antibody aggregates). Line scans were then automatically drawn onto the spots, and the full width at half maximum (FWHM) and the intensity of the spots (cumulative, across the entire line scan) were determined from Lorentzian curve fits to the scans. Panel **B** indicates the spot density, while panel **C** indicates the FWHM, and panel **D** indicates the spot intensity. As expected, the spot density increases over time (panel **B**). No substantial changes can be seen for the FWHM and for the intensity. The aggregates from neuronal cultures are similar to those directly placed on glass coverslips coated with PLL. The box plots indicate the medians with 25^th^ and 75^th^ percentiles of the data. The bars represent the 10^th^ and 90^th^ percentiles and the dots show the 5^th^ and 95^th^ percentiles. The box plots indicate all data points obtained in three independent experiments for every one of the conditions, with the exception of the 10-minute condition, for which we performed 4 independent. For the density estimations we made 2-10 measurements for each experiment; for the FWHM and intensity estimations we measured on average 50-500 spots per experiment (depending on the respective densities).


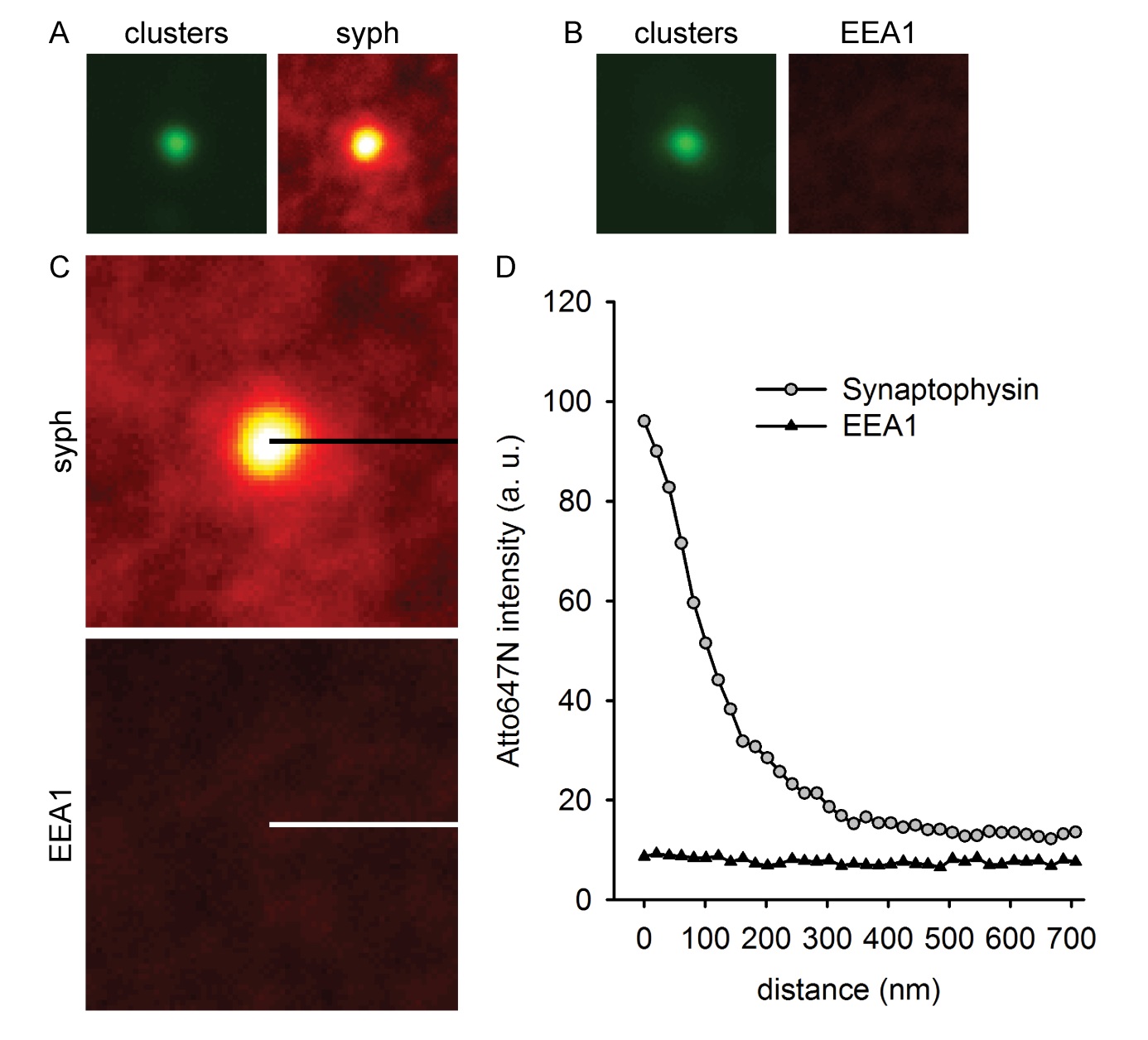


**Supplementary Figure S3. An analysis procedure to determine the colocalization of different proteins of interest to the antibody aggregates.** As shown in Fig. 2, newly exocytosed synaptotagmin is revealed by biotinylated anti-synaptotagmin antibodies, and is sequestered on the plasma membrane with antibody aggregates directed against biotin. These antibodies are labeled using Cy2. The sample is then immunostained, to detect the colocalization of other proteins of interest with the Cy2-labeled antibody clusters. The proteins of interest are detected using Atto647N. To analyze the colocalization, we proceeded as follows. STED and confocal images were first aligned in Matlab, and square regions of interest (71 pixels, or ~1420 nm in size) were automatically generated around the Cy2 spots, using an empirically-determined intensity threshold. These regions of interest were summed, in both the Cy2 and the Atto647N channels, resulting in images such as those in **A**, from a representative experiment for synaptophysin, or in **B**, from a representative experiment for EEA1 (image width = 1420 nm). To then analyze this quantitatively, we drew line scans across the resulting summed matrices, as shown in **C**. The resulting line scans are shown in **D**. The data were later normalized to the baseline (between 600-700 nm from the center), were averaged across experiments, and were shown in Figures 4-7.


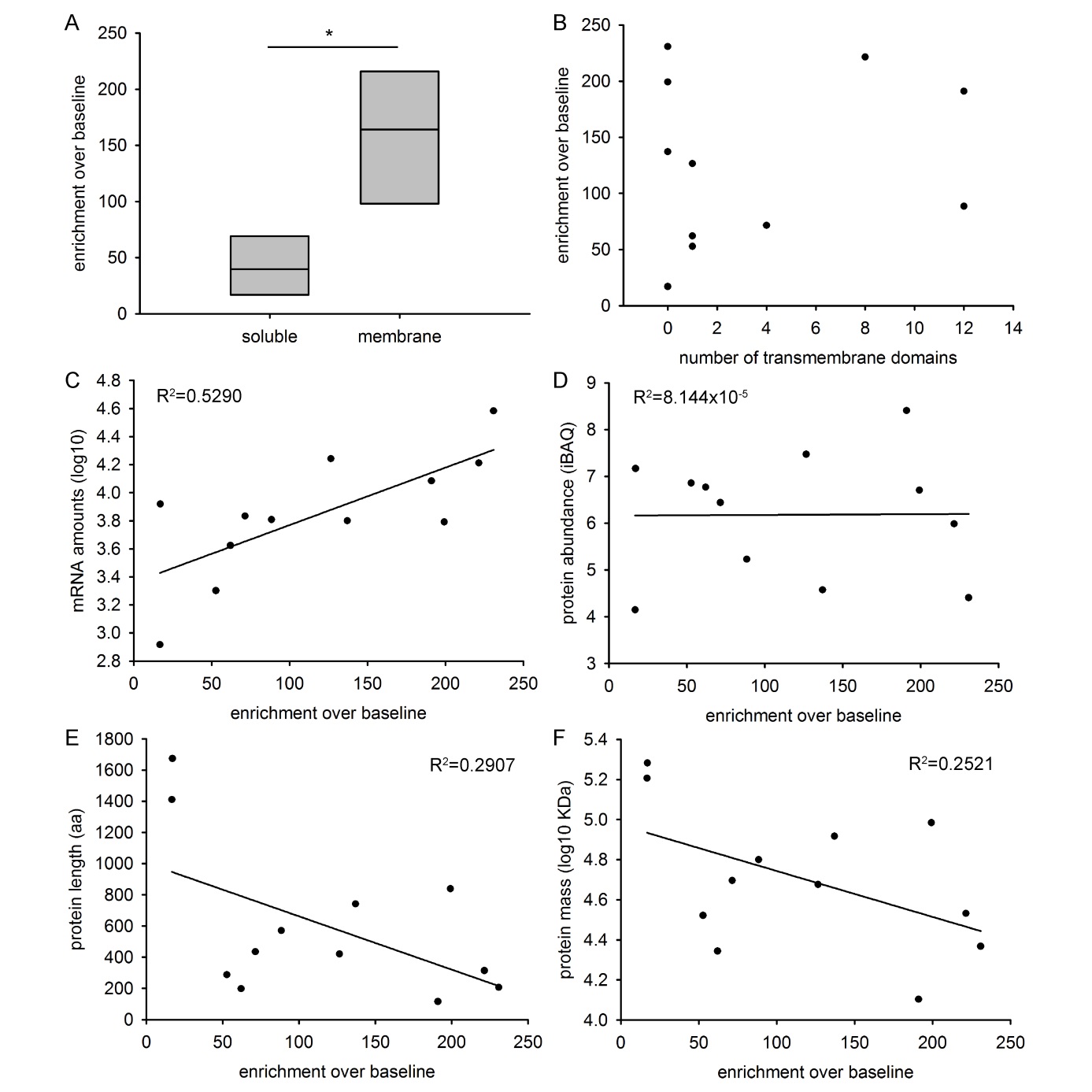


**Supplementary Figure S4. A comparison between the protein enrichment in the vicinity of the antibody aggregates and different neuronal cell biology parameters.** We analyzed the enrichment, over baseline, of each protein in the vicinity of the aggregates, as in Fig. 7. **A**, We compared the enrichment of soluble or membrane proteins. The difference is significant (p=0.016, Mann-Whitney test). **B**, No apparent correlation to the number of transmembrane domains is seen. **C**, A significant correlation to the mRNA amounts of the different genes, as measured in the brain^1^ is observed (p<0.01). Similar results were observed for mRNA amounts from mouse hippocampi^2^. **D**, No correlation could be observed to the protein amounts, as measured in the brain^3^. Similar results were observed for protein amounts estimated in hippocampal cultures^4^. **E-F**, A negative correlation trend is observed for the protein length (E, expressed in number of amino acids, aa) and mass (F), but this is not sufficient for statistical significance (p=0.07 and 0.96 for E and F, respectively). N = 12 analyzed proteins, for all panels.


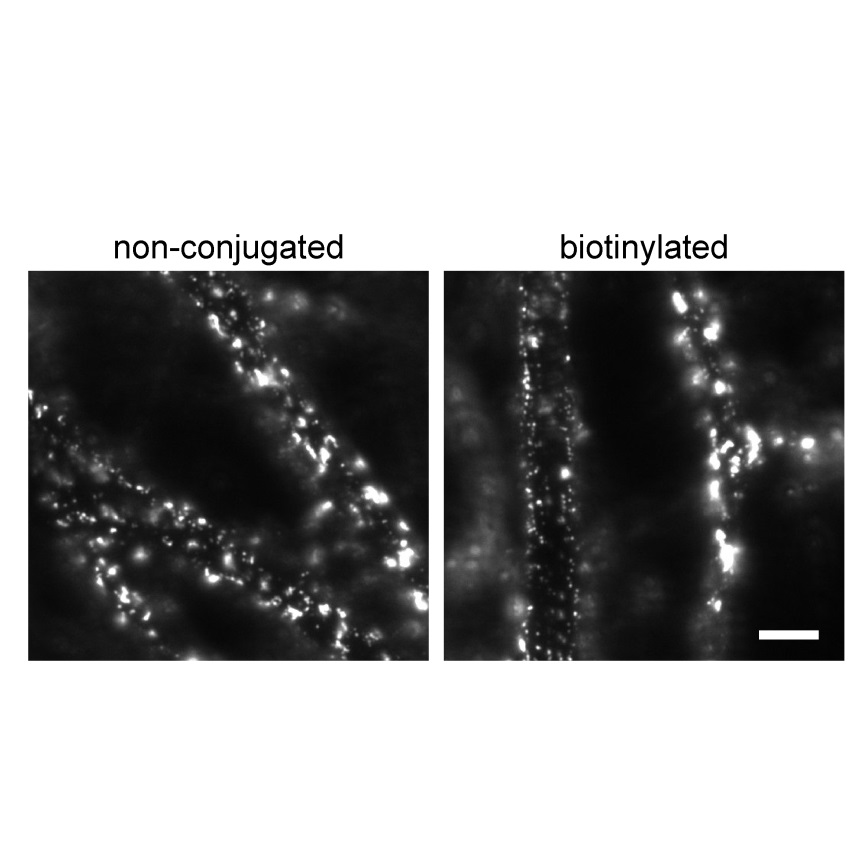


**Supplementary Figure S5. Non-specific binding of streptavidin-labeled beads.** Neurons were incubated with non-conjugated (**left panel**) or biotinylated (**right panel**) anti-synaptotagmin antibodies. The fluorescent beads bind to both samples, indicating strong non-specific binding. Scale bar = 5 μm.

**Supplementary Table S1:** Antibodies used for Immunocytochemistry

| **primary antibody (clone)** | **target protein** | **species** | **Company (cat. number)** | **dilution** | **secondary antibody** | **dilution** |
| --- | --- | --- | --- | --- | --- | --- |
| For surface blocking | | | | | | |
| Anti-syt (604.2) | Synaptotagmin 1 lumenal domain | mouse | Synaptic Systems (#105 311) | 1:20 | - | - |
| For synaptotagmin retention | | | | | | |
| Anti-syt BT (604.2) | Synaptotagmin 1 lumenal domain biotinylated | mouse | Synaptic Systems (#105 311BT) | 1:20 | Ab-aggregates of goat anti-biotin and donkey anti-goat Cy2 | ~ 1:65 and  ~ 1:45 |
| For immunolabeling | | | | | | |
| Anti-syt 1/2 | Synaptotagmin 1/2 cytoplasmic domain | rabbit | Synaptic Systems (#105 002) | 1:1000 | Goat anti-rabbit Cy3 (Dianova #115-165-146) and Goat anti-rabbit Atto647N (Rockland #611-156-122S) | 1:100 and 1:500 |
| Anti-AP 2 | Adapter protein 2 (mu subunit) | rabbit | Abcam (ab75995) | 1:50 | Goat anti-rabbit Atto647N (Rockland  #611-156-122S) | 1:500 |
| Anti-Clathrin | Clathirn (heavy chain) | rabbit | Abcam (ab21679) | 1:500 |  |  |
| Anti-CSP α | Cysteine string protein α | rabbit | Synaptic Systems (#154 003) | 1:500 |  |  |
| Abti-EEA 1 | Early endosomal antigen 1 | rabbit | Synaptic Systems (#237 002) | 1:1000 |  |  |
| Anti- SNAP 25 | Synaptosomal-associated protein 25 | rabbit | Synaptic Systems (#111 002) | 1:100 |  |  |
| Anti-SV 2a | Synaptic vesicle glycoprotein 2a | rabbit | Synaptic Systems (#119 002) | 1:100 |  |  |
| G96 | Synaptophysin | rabbit | Reinhard Jahn | 1:500 |  |  |
| Anti- Synatxin 1b | Syntaxin 1b | rabbit | Synaptic Systems (#110 402) | 1:500 |  |  |
| Anti-VAMP 2 | Synaptobrevin 2 | rabbit | Synaptic Systems (#104 202) | 1:500 |  |  |
| Anti-vATPase | Vacuolar proton pump | rabbit | Synaptic Systems (#109 002) | 1:50 |  |  |
| Anti-vGlut 1/2 | Vesicular glutamate transporter 1/2 | rabbit | Synaptic Systems (#135 503) | 1:100 |  |  |
| For live immunolabeling | | | | | | |
| Anti-syt Oyster550 (604.2) | Synaptotagmin 1 lumenal domain | mouse | Synaptic Systems (#105 311C3) | 1:100 | - | - |

**References**

1. Gonzalez, C. *et al.* Ribosome profiling reveals a cell-type-specific translational landscape in brain tumors. *J. Neurosci.* **34,** 10924–36 (2014).

2. Kadakkuzha, B. M. *et al.* Transcriptome analyses of adult mouse brain reveal enrichment of lncRNAs in specific brain regions and neuronal populations. *Front. Cell. Neurosci.* **9,** 63 (2015).

3. Fornasiero, E. F. *et al.* Precisely measured protein lifetimes in the mouse brain reveal differences across tissues and subcellular fractions. *Nat. Commun.* **9,** 4230 (2018).

4. Schanzenbächer, C. T., Sambandan, S., Langer, J. D. & Schuman, E. M. Nascent Proteome Remodeling following Homeostatic Scaling at Hippocampal Synapses. *Neuron* **92,** 358–371 (2016).
